# Medical Opinion and Movement

**Published:** 1908-05-09

**Authors:** 


### Medical Opinion and Movement.

PEROXIDE OF HYDROGEN probably deserves a much more extended employment in surgical treatment than it has hitherto received, at any rate in this country. Its germicidal and cleansing properties have been known for some time to aurists, and they have made free use of it for instillation in cases of otitis media. In France it has increased enormously in popularity among surgeons, and is used largely as an antiseptic wash and dressing for suppurating wounds and ulcerous surfaces. The annual return showing the quantities of drugs used in the various hospitals and infirmaries of Paris during the year indicate a great increase in the consumption of hydrogen peroxide. A serious deterrent to its more general use has probably consisted in its instability and consequent loss of efficacy on keeping. This is not, however, an insuperable difficulty, in view of the excellent qualities which are otherwise claimed for it. Dr. Hovenden has recently reported some very satisfactory results he has obtained in cases of carbuncle and other severe forms of localised inflammation. He used compresses soaked in hydrogen peroxide and cold water, 1 in 3, and describes the effect as remarkable. In one case the condition of inflammation had become formidable in spite of free incisions and antiseptic applications, but it rapidly subsided on the application of these compresses.

WE have already referred to the promising reports on the treatment of epidemic cerebrospinal meningitis with a serum which has been prepared at the Rockefeller Institute. A further report has recently been issued by Dr. Simon Flexner, medical director of the institute. Details of the experimental research are given by which the serum was eventually obtained, and also the results which have attended its use. By inoculation experiments on guinea-pigs it was found that the meningococcus produces effects on these animals different from those in man, but the lesions in monkeys closely resemble those in man, both macroscopically and microscopically. As in the case of diphtheria antitoxin the horse was used for purposes of immunisation. Only a small dose of 5 cubic centimetres was administered at first, but later this was increased to 10 and 15 c.c., and even larger doses; the injections are made directly into the spinal canal. The first trial of the serum was made during an epidemic in Ohio, where it was used in 40 cases, with 29 recoveries and 11 deaths—a mortality of 27.5 per cent. The serum has since been used in New York, Boston, Philadelphia, Belfast, and elsewhere, with the same satisfactory results. Altogether 130 cases have been treated with the serum, of which 95 have recovered and 35 died, showing the same mortality as in the first series—27.5 per cent. In the last epidemic in New York there were 5,000 cases with a mortality of 70 per cent., so that the use of the serum has resulted in a substantial reduction of the fearful mortality which usually attends the disease.

LICHTENBERG has attempted to summarise the literature dealing with post-operative pulmonary complications, a subject on which much has been written of late years, and one, too, that is as interesting to the special surgeon as to the general practitioner, who may be called upon at any time to give an anæsthetic. In reading over the conclusions drawn by the author one cannot but be struck with the comparative immunity from such pulmonary complications which is claimed for patients who have been narcotised under the scopolamine-morphine method. A full statistical account is difficult to get, and need not be insisted upon; but the author gives a list of 839 patients anæsthetised with the scopolamine-morphine-chloroform method, with a total percentage of 0.7 per cent. post-operative pneumonias, none of them fatal. In this respect the very excellent results recently obtained in America by the so-called Abbot method, in which the scopolamine is replaced by the less dangerous hyoscine, are worth noting, although the author has not taken them into account, being apparently unacquainted with much of the work that has been done on this subject in England and America. In his first bibliography, for example, no English name appears, and the summary cannot therefore be accepted as showing much more than the results obtained by Continental, and especially by German, workers. As such it is not without value, and the critical discussion of the probable causative factors in post-operative broncho-pneumonia is especially worth consideration.

AS primary cause of these complications, Wolff of Königsberg assumes "a toxic action of the anæsthetic itself." Some authorities have tried to show that the hypersecretion which occurs after ether narcosis is purely pathological, and that it agrees with inflammatory exudates generally in possessing a relatively large quantity of serum globulin and serum albumin. This exudate considerably limits the action of the lungs, and almost negatives the "bactericidal action" of the lung epithelium—an action which is by no means proved to exist, notwithstanding Chapman's experiments. All these theories have at present only an academic interest, and for the practitioner the important point is how to prevent such unfortunate complications. The value of preliminary morphine injections has already been referred to, and anyone who has compared the statistics of the "open" and "closed" methods of etherisation will agree that the former is far preferable to the latter. In Germany the open method is not much used, and the small, cramped masks that are in vogue have doubtless influenced the unfavourable statistics which are given. The average practitioner, however, who gives his patients ether on the open Skinner, carefully and patiently, never hurrying the process to "save time" or to "get the patient under quickly," will not be troubled by much post-operative broncho-pneumonias in his practice. In a few cases—such as suppurative peritonitis and cases of strangulated hernia—such complications will occur when the anæsthetic has been in the best possible hands, but in ordinary cases they ought to be preventable by careful attention to the details of the anæsthetic—an attention which, it may be added, one observes much more frequently in English hospitals than in Germany.

EMPYEMA after pneumonia is a subject of perennial interest. Dr. McCrae, of the Johns Hopkins Hospital, states that the statistics from all hospitals indicate that pneumococcal empyema is on the increase. He considers empyema in the vast majority of instances is a complication rather than a sequel of pneumonia. In view of recent observations, which show that the pneumococcus is to be found in the pleura in 100 per cent. of pneumonia cases, Dr. McCrae is rather surprised that empyema is not more frequent. Of 805 recent cases of pneumonia at the Johns Hopkins Hospital empyema occurred in 29 —*i.e.*, 3.6 per cent. In nearly every case the attack of pneumonia was serious. Dr. McCrae states that in no instance was it possible to determine the onset of the empyema. Apart from physical signs, the first thing that attracted notice was continuance of the fever; in only two of the 29 cases was the temperature normal for 24 hours or more. The pulse showed much variation and nothing characteristic. There was invariably a leucocytosis, but the leucocyte count was of no great value in diagnosis. A feeling of resistance to the palpating hand was an important physical sign and correctly suggested fluid when other signs were all against it. In half the cases of empyema vocal fremitus was retained to a greater or less degree, and the presence of this sign often led to delay in exploration. The signs were thus anomalous throughout the series. The author insists on the necessity for using the needle again and again in suspected cases of concurrent empyema and pneumonia. The amount of fluid may be slight, and we cannot depend on the ordinary signs of fluid in the thorax. The experience of English hospitals will endorse Dr. McCrae's view of the great importance of a repeated use of the exploratory syringe in all cases of pneumonia wherein there is a suspicion of the presence of pus.

THE subject of Pancreatitis is dealt with in an able article by William J. Mayo in the April issue of the *New York State Journal of Medicine*. Investigations by W. J. and C. H. Mayo in December last showed that in 2,200 operations upon the gall-bladder and biliary passages the pancreas was coincidently affected 141 times, while in 168 operations for pancreatic disease the condition was associated with gall-stones in 81 per cent. of cases. The author lays great stress upon the necessity for examination of the fæces in the diagnosis of pancreatic lesions. He says, "Frequent large light-coloured, greasy motions without jaundice are indicative of pancreatitis." Of Cammidge's test he says, "it is slow and laborious, and has been more successful in the hands of its originator than with others. Our experience with it, while not extensive, seems to bear out much of his claims for it." When gall-stones are associated with chronic interstitial pancreatitis, operation is attended by much increased liability to hæmorrhage. Mayo, for this reason, gives calcium chloride or lactate before and after operation; but he doubts the value of this measure. He emphasises the necessity for clearing out all the calculi, especially from the common duct, and thoroughly dilating the duct with a malleable probe; whereas Robson found that 60 per cent. of his cases of stone in the common duct had pancreatitis, the brothers Mayo found but 18.6 per cent. W. J. Mayo's clinical description of acute pancreatitis is vigorous: "Acute pancreatitis has sudden onset and is ushered in by agonising pain in the upper abdomen, with collapse followed by extreme prostration. The pulse becomes quick, there is some elevation of the temperature, with nausea, vomiting and rapid abdominal distension. The acuteness of the symptoms suggests obstruction which is belied by the ability to secure the passage of flatus. The patients are usually elderly, obese, and often have an alcoholic history."

AN interesting article in *La Clinique* by Levy-Bing, summarises the history of the micro-organism of syphilis from its discovery in 1905 by Schaudinn and Hoffmann, and the reasons for which its name has been changed from spirochæta pallida to treponema pallidum. A succinct account of the methods used in preparing material for microscopical examination, and of the work done on the life history of the micro-organism, form the bulk of the monograph, but short paragraphs are introduced on the relation of the micro-organism to the known pathology of syphilis, and on the effects of mercurial and of arsenical treatment upon the organism. The author draws the following conclusions based upon the matter contained in his article. 1. The Tr. pallidum is found in all classes of syphilitic lesions, and is present in pure culture when the lesion is not in communication with the surface. Failure to find the organism is the result of imperfect technique. 2. The Tr. pallidum is found in syphilitic lesions only. 3. The Tr. pallidum is found in such places as a study of the pathology of syphilis would point to its being found. 4. Mercurial treatment, in most cases, causes a rapid disappearance of the Tr. pallidum from the lesions. 5. Thus in spite of failure to cultivate the organism it is right to consider the Tr. pallidum as the specific cause of syphilis.

LINDENSTEIN has tabulated 500 cases of lumbar anæsthesia done at the Nurnberger Städtischen Krankenhause, and gives interesting particulars of the methods which have been used and their respective merits. Novocain has here, as generally in Germany and Austria, almost entirely replaced stovain and tropacocain. It is used in the form of a sterilised 5 per cent. solution, 2 c.cm. of which are injected. The patient is allowed to lie flat, no elevation of the pelvis or lowering of the head being considered necessary. All the cases were satisfactory, both from an operative and from an anæsthetic point of view. A full *résumé* of the after-effects is given. In

6 to 8 per cent. of cases the patients suffered from transient headache and throbbing in the ears after the operation; in a few cases there was persistent vomiting, lasting for several days. In two cases there was partial paralysis of single nerves. The author considers the method unsuitable for anæmic patients, and especially for hysterical female patients.

---

PROFESSOR ALFRED STIEDA of Königsberg has paid special attention to certain relatively rare post-operative complications in cases of surgical interference in gastric and abdominal conditions. In operations on the stomach three serious complications are always to be feared. These are peritonitis, atony, and hæmorrhage. The causes of the first are to be sought for in sepsis, perforations, or cutting through of sutures; but the author remarks that it is possible for peritonitis to result without a perforation or other cause being demonstrable at the autopsy. In such cases the explanation is probably to be found in the fact that sepsis may spread through the stomach walls by percolation without any manifest perforation existing. Such a possibility appears to be by no means proved to exist, and one prefers to look upon such peritonitis as really caused by factors which have escaped observation at the post-mortem. Post-operative hæmorrhage, which shows itself as profuse hæmatemesis, is probably always due to bad operative methods, the slipping or cutting out of ligatures, and extensive damaging of the gastric mucosa by handling, instruments, etc. Gastric atony, on the contrary, is usually an unavoidable complication, against which the best surgery cannot safeguard the patient. The author, in addition, draws attention to a little-known complication after operations on the stomach—persistent and fatal vomiting, for which no cause can be discovered at the autopsy. This rare complication is possibly due to increased reflex stimulation, and treatment so far has been useless to save the patients.

---

IN cases of inoperable cancer of the cervix the attendants as well as the patients are usually distressed by the fœtid odour that emanates from the large ulcerating surface, and that seems to overcome every attempt to suppress it. Various methods have been suggested to get rid of this smell. from the administration of remedies internally to surgical operations, such as scraping and removal of part of the fungating mass; but few of them are of such a nature that they can be easily and readily applied by the practitioner. The advice recently given by Gellhorn is therefore worth attention, as it appears to be easy to follow. This author recommends the use of acteone, which is perhaps better known as spiritus pyro-aceticus ($C_3H_6O$) in such distressing cases. The patient is anæsthetised, and the ulcerating surface is well curetted. Thereupon the acteone is applied to the surface of the mass, which has been dried as far as possible, by means of swabs. The action of the acetone is to harden the surface of the sore, and the anæsthetic is only necessary in cases where it is desirable to curette away the sloughs, a preliminary which is always advisable. Afterwards the acetone is applied from time to time, no anæsthetic being required. The results of the treatment are most satisfactory, the foul odour disappearing completely. The applications of acetone have no curative effect whatever on the growth itself.

---

THE pupils of the late Professor Mikulicz of Breslau have recently issued a memorial Festschrift in honour of their master. The Festschrift appears as a supplement to the *Mitteilungen aus den Grenzgebieten der Medizin und Chirurgie*, a work which to a large extent owed its inception to the late Professor Mikulicz, and to which he was, literally up to the day of his death, a voluminous and able contributor. As a contribution to modern surgery, the "Gendenkschrift" is worth careful attention. It includes 30 articles, dealing with widely different subjects, but each one on some subject in the domain of surgery upon which the master himself has shed a ray of light, and into which he had personally encouraged further research by his pupils. Samter of Königsberg deals with the operative treatment of large anal prolapses in which he discusses colopexy, rectopexy, Douglas's operation, and resection. Tietze of Breslau describes three cases of bone implantation; Martin sums up the results obtained at the Breslau clinic in operations on the stomach; Heule has a chapter on plastic operations on the nose; Kümmel of Heidelberg one on general pyæmia due to otitis media; and Niche writes on the casualty stations and their statistics in Breslau; Zondek's article on the surgical treatment of chronic nephritis; Hübener's on typhoid metastases; Gottstein's on bronchoscopy; and Chlumsky's on enterorrhaphy are really monographs on special subjects which contain much that is new and original, and which will appeal to every surgeon.

---

THIS enumeration will show how well the Breslau school has worked at the subjects "set by the master," for everyone of these subjects was one in which Mikulicz was himself interested, on which he wrote, and to the study of which he brought new ideas and the valuable experience his large operative practice had given him. The result is that the whole book is a most valuable addition to modern surgical literature, and that it is in fact an epitome of the teaching of the Breslau school in several special departments. The various articles show much patient research, wide reading, and careful and painstaking verification, so far as was possible, of details. It is scarcely a work that will interest the general practitioner (except in so far that it is an excellent illustration of the advance of surgery in certain departments, especially in pulmonary and intrathoracic surgery), but to the practising surgeon it offers a remarkably rich field, and its perusal will be as interesting as it is instructive. For to a great extent the pupils have not forgotten the master's caution that even in scientific articles it is not only necessary to write clearly and accurately, but it is equally necessary to cultivate an attractive and readable style.

